# Mortality in patients with HIV-1 and tuberculosis co-infection in Rio de Janeiro, Brazil - associated factors and causes of death

**DOI:** 10.1186/s12879-017-2473-y

**Published:** 2017-05-30

**Authors:** Rodrigo Otavio da Silva Escada, Luciane Velasque, Sayonara Rocha Ribeiro, Sandra Wagner Cardoso, Luana Monteiro Spindola Marins, Eduarda Grinsztejn, Maria Cristina da Silva Lourenço, Beatriz Grinsztejn, Valdiléa Gonçalves Veloso

**Affiliations:** 1 Instituto Nacional de Infectologia Evandro Chagas, Fundação Oswaldo Cruz, Rio de Janeiro, Brazil; 2 Departamento de Matemática e Estatística, Universidade Federal do Estado do Rio de Janeiro, Rio de Janeiro, Brazil

**Keywords:** Tuberculosis, Mortality, CoDe, Bacterial disease, Tuberculosis, HIV/Aids, Cohort study, Brazil

## Abstract

**Background:**

Tuberculosis is the most frequent opportunistic infection and the leading cause of death among persons living with HIV in several low and middle-income countries. Mortality rates during tuberculosis treatment and death causes among HIV-1/TB co-infected patients may differ based on the immunosuppression severity, timing of diagnosis and prompt initiation of tuberculosis and antiretroviral therapy.

**Methods:**

This was a retrospective observational study conducted in the clinical cohort of patients with HIV-1/Aids of the National Institute of Infectious Diseases Evandro Chagas, Rio de Janeiro, Brazil. All HIV-1 infected patients who started combination antiretroviral therapy up to 30 days before or within 180 days after the start of tuberculosis treatment from 2000 to 2010 were eligible. Causes of death were categorized according to the "Coding Causes of Death in HIV” (CoDe) protocol. The Cox model was used to estimate the hazard ratio (HR) of selected mortality variables.

**Results:**

A total of 310 patients were included. Sixty-four patients died during the study period. Mortality rate following tuberculosis treatment initiation was 44 per 100 person-years within the first 30 days, 28.1 per 100 person-years within 31 and 90 days, 6 per 100 person-years within 91 and 365 days and 1.6 per 100 person-years after 365 days. Death probability within one year from tuberculosis treatment initiation was approximately 13%. In the adjusted analysis the associated factors with mortality were: CD4 ≤ 50 cells/mm3 (HR: 3.10; 95% CI: 1.720 to 5.580; *p* = 0.00); mechanical ventilation (HR: 2.81; 95% CI: 1.170 to 6.760; *p* = 0.02); and disseminated tuberculosis (HR: 3.70; 95% CI: 1.290 to 10.590, *p* = 0.01). Invasive bacterial disease was the main immediate cause of death (46.9%).

**Conclusion:**

Our results evidence the high morbidity and mortality among patients co-infected with HIV-1 and tuberculosis in Rio de Janeiro, Brazil. During the first year following tuberculosis diagnosis, mortality was the highest within the first 3 months, being invasive bacterial infection the major cause of death. In order to successfully intervene in this scenario, it is utterly necessary to address the social determinants of health contributing to the inequitable health care access faced by this population.

## Background

In high burden areas such as Brazil and other developing countries, tuberculosis (TB) remains a serious public health concern [1]. Throughout the last decade the incidence of TB and its related mortality have steadily declined in Brazil. Nevertheless, this downward trend is not observed among HIV-1 infected patients [2, 3].

TB is the most frequent opportunistic infection and the leading cause of death among persons living with HIV (PLHIV) in several low and middle-income countries (LMIC), particularly among individuals with advanced HIV disease [4]. Of the 1.5 million deaths attributed to TB in 2013, 24% were among PLHIV [1, 2].

Causes of death among HIV/TB co-infected patients may differ based on the immunosuppression severity, timing of diagnosis and prompt availability of TB and antiretroviral therapy (cART). It is well known that TB can directly contribute to early mortality in PLHIV; however, other infections and comorbidities may also play an important role [5–7]. Therefore, additional insights on the causes of death in HIV/TB co-infected patients are needed, particularly in high burden settings.

This study aims to describe short and long-term mortality rates, its associated factors as well as causes of death in HIV-1/TB patients in a cohort of PLHIV in Rio de Janeiro, Brazil.

## Methods

### Design

This retrospective observational study was conducted at the Evandro Chagas National Institute of Infectious Diseases-Oswaldo Cruz Foundation (INI-Fiocruz) in Rio de Janeiro, Brazil. INI-Fiocruz has been a reference center for care, research, and training for HIV-1/AIDS since 1986, being one of the largest providers of primary, specialty, and tertiary care for HIV infected individuals in Rio de Janeiro State. A longitudinal observational clinical database has been maintained on patients receiving care for HIV-1 at INI-Fiocruz since 1998. The details of this cohort have been previously described and published elsewhere [8, 9].

### Study population

The participants included in this analysis are part of the INI cohort. All included patients presented to care when symptomatic and were then investigated for tuberculosis as well as for the opportunistic infections, according to their clinical presentation. As of December 2010, approximately 4000 patients were included in the HIV-1/AIDS cohort at INI-Fiocruz. Patients who started cART within 30 days before and up to 180 days after TB treatment initiation, in the period between January 2000 and December 2010, were included in this analysis. Patients with prior exposure to any ARV and those who started follow-up at the INI-Fiocruz cohort more than 60 days after TB treatment initiation were excluded. All patients included in the study were followed until December 31th, 2013 or until death, whichever occurred first.

### Data collection and management

For this analysis, besides the data already available from the INI-Fiocruz HIV-1 database, further data was collected from medical, laboratory and image records and registered on standardized forms. The “Coding of Death in HIV” (CoDe) protocol was used to standardize causes of death report [10, 11].

### Anti-TB and antiretroviral therapy (ART)

ART was offered to patients as standard of care and was adjusted over time based on the Brazilian National Guidelines. Co-trimoxazole prophylaxis was prescribed according to national guidelines. The first line anti-tuberculosis regimen during most of the study period was the combination of rifampicin, isoniazid and pyrazinamide for the first 2 months of therapy (“intensive phase”), followed by rifampicin plus isoniazid for the last 4 months of therapy (“continuation phase”). If there was evidence of central nervous system involvement, the “continuation phase” of treatment was extended to 7 months. From July 2009 onwards, ethambutol was added to the “intensive phase” regimen. TB treatment was adjusted in cases of severe adverse events, drug resistance and need of cART regimens that precluded the use of rifampicin. Data on history of isoniazid preventive therapy (IPT) was captured from the cohort database.

### Definitions

For this study, the following definitions were used:

cART was defined as the combination of two nucleoside/nucleotide analogue reverse transcriptase inhibitors (NRTI) with a non-analogue nucleoside reverse transcriptase inhibitor (NNRTI), an integrase strand transfer inhibitor (INSTIs), or a protease inhibitor (PI), enhanced or not with ritonavir (RTV), or two PIs.

Confirmed TB was defined as the presence of a positive culture specimen identified as *Mycobacterium tuberculosis* (MTB) from sputum, lymph node or any other sterile site or MTB identified in a sputum sample using a molecular test (The Amplified *Mycobacterium Tuberculosis* Direct Test [E-MTD®; Gen-Probe, San Diego, CA, USA] or Genotype® MTBDR line probe assay [Hain Lifescience GmbH, Nehren, Germany]). Probable TB was defined as any clinical-radiological suspect case of lung or extrapulmonary disease in which the consulting physician decided to start TB treatment [12]. The date of TB diagnosis was defined as the date of TB treatment initiation.

Pulmonary TB was defined as disease limited to the lungs; extrapulmonary TB as disease restricted to a single-organ system (excluding lungs); disseminated TB as TB in at least two noncontiguous organ systems (one of which could be lungs or pleura) or when a chest X-ray or Computed tomography (CT) scan showed miliary infiltrate or if MTB had been isolated from blood or bone marrow or when there was spleen or liver involvement shown by abdominal ultrasound or CT scan or when bone marrow histopathology yielded a Zihel Nielsen positive acid fast bacilli or granuloma [13–15]. MTB isolates resistant to at least rifampicin and isoniazid were defined as multi-drug resistant [16].

The definition of a *Mycobacterium tuberculosis* immune reconstitution inflammatory syndrome (MTB-IRIS) episode was the identification of this diagnosis description in the medical record during antituberculosis and antiretroviral (ARV) treatment.

Invasive bacterial disease was defined as the inbreak of normally sterile tissue, fluid or body cavity by pathogenic or potentially pathogenic micro-organisms, such as pneumonia and meningitis, and/or sepsis [17].

AIDS-defining illnesses were classified according to the Centers for Disease Control 1993 definitions [18]. Concomitant AIDS-defining illness was considered when occurred within 30 days before or after TB treatment initiation.

Early death was defined as death occurring within 90 days after TB treatment initiation.

### Study variables

The following variables were explored: age at TB diagnosis, gender, race, years of schooling, CD4 T lymphocyte count at time of TB diagnosis (categorized as ≤50 cells/mm^3^ and >50 cells/mm^3^) collected up to six months before the diagnosis of TB. This cut off was elected once it is used to define timing of ART initiation in the STRIDE study [19]. Other variables of interest were: discontinuation of rifampicin prior to the end of TB treatment, presence or not of AIDS-defining illness concomitant to the episode of TB within 30 days before or up to 30 days after the start of TB treatment, hospitalization within one year following TB treatment initiation, use of mechanical ventilation during hospitalization, change in ARV regimen due to toxicity, co-trimoxazole use alongside TB and cART for PCP prophylaxis, prior IPT Isoniazid preventive therapy use, presence or absence of an episode of MTB-IRIS, and time of death (≤ 90 days or >90 days).

### Statistical analysis

Continuous variables were reported as mean ± standard deviation or median (range) and discrete variables were reported as absolute and relative frequency. Pearson’s χ2 or Fisher’s exact test were used for qualitative comparisons and Mann-Whitney test for quantitative comparisons. To characterize the study population, a descriptive analysis of all variables considered in the study was performed. Patients, both dead and alive were compared using Pearson’s χ2 test or Fisher’s exact test for categorical variables and the non-parametric Mann-Whitney test for quantitative variables.

The Kaplan-Meier curve [20] was plotted to estimate the survival probability after TB treatment initiation and the log-rank test was calculated for univariate analysis to compare the probability of survival amongst the different clinical presentations of TB.

The time dependent Cox proportional-hazard model was used for uni and multivariate analyses after verification of the proportional-hazard assumption using the Schoenfeld’s test. Models were stratified by the variables that did not respect the proportional assumption. Variables that had a 20% significance level in the univariate analysis were selected for multivariate model. A backward procedure was used to remove variables one by one with the largest *p*-values in order to obtain the final fitted model. Variables with borderline *p*-values (*p* ≤ 0.10) and variables that exhibited a significant effect (Hazard Ratio [HR] > 2.0) were retained in the model [20, 21].

All analyses were performed using R software version 3.1.1 (R Core Team, 2013).

## Results

**Fig. 1:**
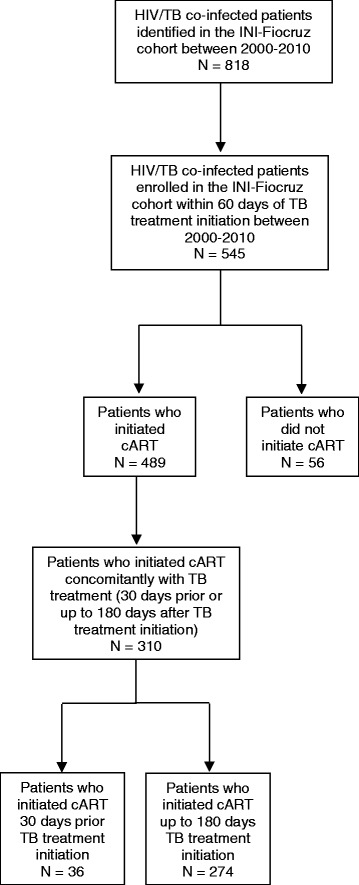
Flowchart of inclusion/exclusion criteria for HIV-1/TB co-infected patients, INI-Fiocruz Cohort, 2000–2010

From 2000 to 2010, a total of 818 patients with TB were identified in the database; 310 patients fulfilled all inclusion criteria for this analysis (Fig. 1). Patients were followed for 1.568 person/years; median follow up was 55.5 months (IQR: 35.5–79.6).

Table 1 summarizes patient’s demographics and clinical characteristics. The median baseline number of CD4 T lymphocytes count at TB diagnosis was 91 cells/mm^3^ (IQR: 42.0–215.0); 120 patients (38.7%) had a CD4 T lymphocytes count ≤50 cells/mm^3^ and the median baseline viral load was 5.2 log (IQR: 4.5–5.7).

**Table 1:** Demographic and clinical characteristics of HIV-1/TB co-infected patients according to vital status, INI-Fiocruz cohort, 2000–2010

	Total	Alive	Dead	*p value*
Patients (n; %)	310 (100)	246 (79.4)	64 (20.6)	
Sex (n; %)				
Male	233 (75.2)	185 (75.2)	48 (75.0)	1.000
Female	77 (24.8)	61 (24.8)	16 (25.0)	
Age at TB diagnosis (years) (median; IQR)	36.9 (30.2–43.3)	36.5 (30.1–42.5)	37 (30.9–45.7)	0.243
Race (n, %)				
White	111 (35.8)	86 (35.0)	25 (39.0)	
Black	79 (25.5)	67 (27.2)	12 (18.8)	0.373
Mixed Black	119 (38.4)	92 (37.4)	27 (42.2)	
Unknown	1 (0.3)	1 (0.4)	-	
Education (n; %)				
Up to 4 years	132 (42.6)	102 (41,5)	30 (46,9)	
5–8 years	86 (27.7)	66 (26.8)	20 (31.2)	0.307
> 8 years	92 (29.7)	78 (31.7)	14 (21.9)	
CD4 T cell count at TB diagnosis (cells\mm^3^) (median; IQR)	91 (42.0–215.0)	96 (45.0–220.5)	69.5 (34.0–146.0)	0.079
HIV-1 RNA (log_10_ copies/ml) (median; IQR)	5.2 (4.5–5.7)	5.1 (4.5–5.7)	5.4 (4.8–5.7)	0.389
Interval between TB treatment and cART initiation (days) (Median; IQR)	33 (17.5–61.0)	35 (25.0–61.8)	25.5 (0.8–57.0)	0.018
Number of ARV changes due to toxicity (median; IQR)	0 (0.0–0.0)	0 (0.0–0.0)	0 (0.0–0.8)	0.574
PCP prophylaxis (n; %)				
No	14 (4.5)	13 (5.3)	1 (1.6)	0.315
Yes	196 (95.5)	233 (94.7)	63 (98.4)	
IPT (n; %)				
No	270 (87)			
Yes	4 (1)			
Unknown	36 (12)			
Clinical presentation of TB (n; %)				
Pulmonary	47 (15.2)	36 (14.6)	11 (17.2)	
Disseminated	227 (73.2)	179 (72.8)	48 (75,0)	0.573
Extrapulmonary, localized	35 (11.3)	30 (12.2)	5 (7.8)	
Unknown	1 (0.3)	1 (0.4)	-	
TB treatment (n; %)				
RHZ	199 (64.2)	160 (65.0)	39 (60.8)	0.643
RHZE	105 (33.9)	81 (32.9)	24 (37.5)	0.589
Others	6 (1.9)	5 (1.1)	1 (1.7)	
Rifampin discontinuation before the end of TB treatment (n; %)	34 (11.2)	24 (9.8)	10 (15.6)	0.471
Hospitalization during the first year after treatment TB (n; %)				
No	120 (38.7)	107 (43.5)	13 (20.3)	
Yes	190 (61.3)	139 (56.5)	51 (79.7)	0.001
Mechanical ventilation (n; %)	20 (6.4)	6 (2.4)	14 (21.8)	<0.001
MTB-IRIS (n, %)	38 (12.3)	29 (11.8)	9 (14)	0.763
Unknown	3 (0.9)	2 (0.8)	1 (1.6)	

*TB* tuberculosis

*ARV* antiretroviral

*cART* combination antiretroviral therapy;

*PCP Pneumocystis jirovecii* pneumonia (formerly called *Pneumocystis carinii*);

*MTB-IRIS Mycobacteryum tuberculosis* related immune reconstitution inflammatory syndrome;

*RHZ* rifampin, isoniazid and pyrazinamide;

*RHZE* rifampin, isoniazid, pyrazinamide and ethambutol;

*IPT* isoniazid preventive therapy

The median time from HIV-1 infection diagnosis to TB diagnosis was 35.5 days (IQR: 9.0–204.0). Thirty-six patients (11.6%) started cART up to 30 days before TB treatment initiation (median time 14.5 days [IQR: 9.0–22.0]), and 274 (88.4%) initiated cART within 180 days following TB treatment initiation (median time 36 days [IQR: 27.0–67.0]) and 15 (5.4%) initiated cART within 2 weeks. The median time between TB treatment and cART initiation was significantly lower among patients who have died than among those who lived (25.5 days [IQR: 0.8–57.0] vs 35.0 days [IQR: 25.0–61.8]) (*p* = 0.018; Table 1). The most frequently prescribed cART regimen was the combination of two NRTI with efavirenz (EFV) (82.9%; *n* = 257/310), followed by the combination of two NRTIs, saquinavir (SQV) and RTV at full dose (6.5%; *n* = 20/310) and the combination of two NRTIs and raltegravir (RAL) (5.5%; *n* = 17/310).

Overall, 64.2% (*n* = 199/310) patients had confirmed TB: 52.6% (*n* = 163/310) based on a positive culture and 11.6% (*n* = 36/310) using E-MTD®. Four patients were diagnosed with multi-drug resistant-Tuberculosis (MDR-TB) (2.5% of all culture confirmed cases), 5 patients had isolated isoniazid resistance (3.1% of all culture confirmed cases) and 3 patients had isolated rifampicin resistance.

In total, 73.2% (*n* = 227/310) patients had disseminated TB, 11.3% (*n* = 35/310) had localized extrapulmonary TB, and 15.2% (*n* = 47/310) had pulmonary TB. The majority of the patients (98.1%; *n* = 304/310) started a rifampin-based TB treatment regimen; 11.2% (*n* = 34/304) of the patients discontinued rifampicin before the end of TB treatment, and this was primarily due to hepatotoxicity (29.4%; *n* = 10/34). The proportion of patients who had a definitive interruption of rifampin did not differ significantly from those who lived and those who died (9.8% vs 15.6%; *p* = 0.471). Nearly 61.0% (*n* = 190/310) of the patients required at least one hospitalization within the first year of TB treatment. A total of 6.5% (*n* = 20/310) of the patients needed mechanical ventilation; 70.0% (*n* = 14/20) of them deceased. MTB-IRIS was reported in 12.3% (*n* = 38/310) (Table 1).

Overall, 12.3% (*n* = 38/310) of the patients had a prior TB episode. In 32.3% (*n* = 100/310), an AIDS-defining illness was diagnosed concomitantly with the TB diagnosis, namely *Pneumocystis jirovecii* pneumonia (13.5%), esophageal candidiasis (5.8%) and neurotoxoplasmosis (5.2%). In addition, 19.4% (*n* = 60/310) had invasive bacterial disease concomitantly with TB (Table 2).

**Table 2:** Aids-Defining illnesses diagnosed concomitantly with TB, *n* = 310, INI-Fiocruz cohort, 2000–2010

*Pneumocystis jirovecii* pneumonia; n (%)	42 (13.5)
Candidiasis, Esophageal (presumptive diagnosis); *n* (%)	18 (5.8)
*Toxoplasma gondii* encephalitis; *n* (%)	16 (5.2)
Cytomegalovirus disease (colitis, esophagitis, disseminated); *n* (%)	6 (1.9)
Histoplasmosis, disseminated; *n* (%)	5 (1.6)
Kaposi’s sarcoma; *n* (%)	5 (1.6)
Cryptosporidiosis; *n* (%)	3 (1.0)
Cryptococcal meningoencephalitis; *n* (%)	3 (1.0)
HIV-1 encephalopathy; *n* (%)	1 (0.3)
Lymphoma; *n* (%)	1 (0.3)

Aids-defining illnesses were defined according to the CDC 1993 definition [15]. A concomitant Aids defining illness was considered when it occurred within 30 days before or after TB treatment initiation

Sixty-four patients (20.6%) died during the study period; the median age at death was 40 years (IQR: 32.0–47.0). The median time from TB diagnosis to death was 193 days (IQR: 57.0–1115.0); 17.2% (*n* = 11/64) and 32.8% (*n* = 21/64) of the deaths occurred respectively during the initial 30 days and within the initial 90 days after TB treatment initiation. The overall mortality rate was 4 per 100 person-years (95% CI: 0.4–34.7). The mortality rate up to 30, from 31 to 90, from 91 to 365 and after 365 days following TB treatment initiation was 44 per 100 person-years (95% CI: 10.2–190.6), 28.1 per 100 person-year (95% CI: 7.3–112.0), 6.0 per 100 person-year (95% CI: 3.0–12.0), and 1.6 per 100 person-year (95% CI: 0.09–2.7), respectively. No statistically significant difference in mortality was found between those with confirmed TB (18.2% of deaths) versus those with probable TB (25% of deaths) (*p* = 0,154).

**Fig. 2:**
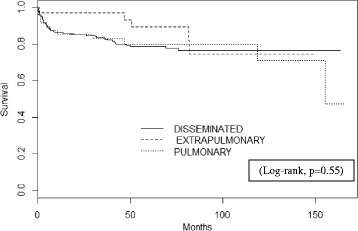
Kaplan-Meier survival curve after TB treatment initiation according to TB clinical presentation, *n* = 310, INI-Fiocruz cohort, 2000–2010

The probability of death within one year from the TB treatment initiation was approximately 13%. The Kaplan-Meier curve shows a higher survival rate among patients who presented with extrapulmonary TB, but no statistical difference was observed in the probability of survival according to TB clinical presentation (log-rank *p* = 0.55) (Fig. 2).

Overall, invasive bacterial disease was the most frequent immediate cause of death (46.9%), followed by AIDS-related illnesses (15.6%). When considering any of the CoDe categories (underlying, immediate and contributing causes of death), TB was associated with 71.4% and 48.8% of the deaths that occurred within the first 90 days and beyond 90 days from TB treatment initiation, respectively. Three deaths were possibly related to MTB IRIS; 1 death occurred within 90 days of TB treatment initiation (cART was initiated 36 days after TB treatment initiation) and 2 deaths occurred more than 90 days from TB treatment initiation (cART was initiated 29 and 31 days after TB treatment initiation) (Table 3).

**Table 3:** Causes of death after TB treatment initiation, *n* = 310, INI-Fiocruz cohort, 2000–2010

Underlying cause of death; *n* (%)				Immediate cause of death; *n* (%)			Contributing cause of death^a^; *n*	
	≤ 90 days *N* = 21	> 90 days *N* = 43		≤ 90 days *N* = 21	> 90 days *N* = 43		≤ 90 days	> 90 days
Aids	18 (85.72)	30 (69.80)	Invasive bacterial disease	11 (52.38)	19 (44.20)	Tuberculosis	12	15
Invasive bacterial disease	1 (4.76)	1 (2.30)	Aids	4 (19.04)	6 (14.00)	Aids	9	18
Renal failure	0	2 (4.70)	Tuberculosis	3 (14.30)	6 (14.00)	Invasive bacterial disease	4	2
Hepatic cirrhosis	1 (4.76)	0	Acute coronary disease	0	1 (2.30)	IRIS	1	2
Hepatitis C, chronic		1 (2.30)	Heart failure	0	1 (2.30)	Renal failure	1	1
Heart failure	0	1 (2.30)	Renal failure	0	1 (2.30)	Hepatotoxicity	0	1
Acute coronary disease	0	1 (2.30)	Hepatic failure	1 (4.76)	0	Hepatic failure	0	2
Chronic obstructive pulmonary disease (COPD)		1 (2.30)	Acute respiratory distress	0	1 (2.30)	Hepatitis B, chronic	0	1
Depression	0	1 (2.30)	Lactic acidosis	0	1 (2.30)	Others	4	15
Violent death	0	2 (4.70)	Suicide	0	1 (2.30)			
Drug abuse		1 (2.30)	Violent death	0	1 (2.30)			
Unknown	1 (4.76)	2 (4.70)	Head trauma	0	1 (2.30)			
			Digestive hemorrhage	1 (4.76)	0			
			Unknown	1 (4.76)	4 (9.4)			

^a^More than one contributing cause of death can be reported.

*IRIS: *immune reconstitution inflammatory syndrome.

In the multivariate analysis, the final model was adjusted for age and gender and was stratified by early death (< 90 days after initiation of TB treatment), once this variable did not respect the proportional assumption. The following parameters were independently associated with higher mortality risk [HR (95% CI)]: (i) CD4 T lymphocyte count ≤50 cells/mm^3^ [3.10 (1.72–5.58); *p* < 0.001], (ii) requirement of mechanical ventilation [2.81; (1.17–6.76; *p* = 0.02)] and disseminated clinical presentation of TB [3.70; (1.29–10.59; *p* = 0.01)].

In the adjusted analysis, patients with a CD4 T lymphocyte count ≤50 cells/mm^3^ exhibited a 3-fold higher mortality rate compared to patients with a CD4 T lymphocyte count >50 cells/mm^3^ (HR: 3.10; 95% CI: 1.720–5.580; *p* < 0.001). Those patients who required mechanical ventilation during a hospitalization exhibited a 2.8-fold increase in mortality compared to patients who did not require (HR: 2.81; 95% CI: 1.170–6.760; *p* = 0.02). Lastly, patients with disseminated TB had an approximately 4-fold increase in mortality compared to patients with localized pulmonary disease (HR: 3.70; 95% CI: 1.290–10.590; *p* = 0.01). Pulmonary TB clinical presentation, although not significant, exhibited an approximately 2-fold increase in mortality rate when compared to extrapulmonary clinical presentation (HR: 2.42; 95% CI: 0.720–8.150; *p* = 0.15). Age and race variables were kept in the final model (*p* ≤ 0.10). Black patients had twice the mortality rate compared to white patients (HR: 2.03; 95% CI: 0.870–4.780; *p* = 0.10). Age at TB diagnosis was associated with an increase of 3% in mortality rate for each year of life (HR: 1.03; 95% CI: 1.000–1.050; *p* = 0.09) (Table 4).

**Table 4:** Crude and Adjusted HR and 95% CI as estimated by Cox proportional hazards regression for death, *n* = 310, INI-Fiocruz cohort, 2000–2010

Variable	Crude HR (CI 95%)	*p* value	Adjusted HR (CI 95%)	*p* value
Sex				
Male	Ref.			
Female	1.54 (0.84–2.80)	0.16		
Education				
Up to 4 years	Ref.			
5–8 years	0.75 (0.41–1.35)	0.33		
> 8 years	1.26 (0.64–2.48)	0.50		
Age at TB diagnosis	1.03 (0.99–1.06)	0.08	1.03 (1.00–1,05)	0.09
Race				
White	Ref.			
Black	1.21 (0.59–2.49)	0.61	2.03 (0.87–4.78)	0.10
Mixed Black	1.22 (0.69–2.16)	0.49	1.68 (0.89–3.16)	0.11
CD4 T cell count at TB diagnosis (cells\mm^3^)				
> 50	Ref.			
≤ 50	2.31 (1.37–3.88)	0.00	3.10 (1.72–5.58)	0.00
Number of ARV change for toxicity	2.12 (0.47–9.55)	0.33		
Clinical presentationof TB				
Extrapulmonary, localized	Ref.			
Disseminated	2.60 (0.97–6.97)	0.06	3.70 (1.29–10.59)	0.01
Pulmonary	1.51 (0.50–5.57)	0.46	2.42 (0.72–8.15)	0.15
Rifampin discontinuation before the end of TB treatment	1.72 (0.83–3.56)	0.15		
Mechanical ventilation (n; %)	2.91 (1.36–6.22)	0.01	2.81 (1.17–6.76)	0.02
MTB-IRIS	0.77 (0.37–1.57)	0.47		

*HR* hazard ratio, *95%CI* 95% confidence interval;

*TB* tuberculosis;

*ARV* antiretroviral;

*MTB-IRIS Mycobacteryum tuberculosis* related immune reconstitution inflammatory syndrome

## Discussion

The results of our study evidence the high morbidity and mortality among patients co-infected with HIV-1 and TB in Rio de Janeiro, Brazil. During the first year following TB diagnosis, mortality was the highest within the first 3 months, being invasive bacterial infection the major cause of death. The 13% mortality within the first year after TB diagnosis found in our cohort is higher than the mortality reported in Argentina but much lower than the mortality described in Eastern Europe [22, 23] and Sub-Saharan Africa [24]. Of note, our patients received highly qualified HIV services including integrated HIV-1/TB care, universal access to cART, opportunistic infections diagnosis and treatment and intensive care management.

The very low median CD4 counts at HIV-1/TB diagnosis highlights that most patients were diagnosed with advanced immunodeficiency. Although some progress on timing of HIV-1 diagnosis was achieved in Brazil, late diagnosis and its consequences remain a major public health issue. Late diagnosis also has a great impact on the timely use of TB chemoprophylaxis. In our cohort, only 4 individuals reported having used IPT. Studies of IPT use in PLHIV in Brazil showed reduced incidence of active TB [25] and improved survival [26] that were incremental to the impact of stand-alone cART.

Although the median time from TB treatment initiation to cART initiation was 36 days, it was not enough to avoid the high rates of early mortality observed in our study. TB diagnosis delay represents a well-known factor associated with poor health outcomes in these patients. Interventions to address barriers to prompt TB diagnosis are essential to curb the high mortality among HIV-1/TB co-infected patients [27, 28]. The recent incorporation of the GeneXpert® technology by the Brazilian Ministry of Health will certainly contribute to reduce the delay for TB diagnosis.

Disseminated TB was the most frequent TB clinical presentation in our study population (73.2%). This can be explained by the more comprehensive definition of disseminated TB used in our study and also the work up performed to accurately diagnose it. Disseminated TB is a common clinical presentation in patients with advanced immunodeficiency and has been associated with increased mortality rates in several studies [29–32].

We cannot exclude the fact that at least some of the diagnosed TB cases within 30 days of cART initiation actually could represent unmasking MTB-IRIS cases. A significant risk of opportunistic infection (OI) persists after cART initiation, especially during the first few months of treatment, and TB is the most frequent of these OIs, particularly in resource-limited settings. This is likely due to a progression of subclinical TB that was present before ART initiation, caused by persisting immunodeficiency or due to unmasking during ART-induced immune recovery [33]. It is of utmost importance to improve screening for OIs, particularly TB prior to ART initiation. The increasing availability of rapid point of care tests for TB and cryptococcosis diagnosis will improve these assessments [34].

Several studies have reported TB as the leading cause of death among patients with HIV-1/AIDS. However, the great majority of them lacked a standardized methodology to assess the causes of deaths [5–7, 35–37]. Using a standardized protocol to ascertain causes of death [10] we were able to demonstrate that invasive bacterial infections is the most frequent immediate cause of death among patients with HIV-1/TB coinfection in our cohort. This is particularly relevant when we consider the early deaths, as previously shown by our group [8] and others [38, 39]. In Brazil, a large proportion of patients die in the hospital and invasive bacterial infections frequently emerge as an end-of-life complication [36, 40]. Patients with HIV-1 are at high risk for nosocomial infections due to immunosuppression, frequent invasive procedures and multispectral antibiotic administration. Although Co-trimoxazole use as *Pneumocystis jirovecii* pneumonia prophylaxis is known to reduce overall mortality [41, 42] and to play a role in the prevalence of overall bacterial infection among HIV patients [43], its usage in our study has not influenced the overall mortality. The ongoing high burden of bacterial infections in our study population indicates an urgent need for additional approaches to prevent bacterial infections, such as expansion of influenza and pneumococcal vaccination coverage, particularly in the Brazilian context, where this strategy is not yet fully implemented.

Severe immunodeficiency remained as an independent associated factor with mortality in our study population, highlighting the need for earlier HIV-1 diagnosis and prompt initiation of cART. Early initiation of cART has been shown to decrease the incidence of AIDS related and non AIDS related complications [44–46]. In the STRIDE study, higher mortality rates were observed amongst individuals with CD4 counts lower than 50 and late cART initiation [19].

Despite of the major advances achieved in the last decade, social inequalities still prevail in Brazil and health disparities may explain the two-fold mortality rate differences in our study for black patients when compared to white [47]. According to the 2010 Brazilian census the average income of black individuals was nearly half of the average income of white and mortality rates were higher when compared to white adjusted for gender and age [48]. Tuberculosis is a disease strongly related to poverty. Inadequate nutrition, poor living conditions, and limited access to adequate health services for early diagnosis and TB treatment are associated with worse outcomes.

A major strength of our study is its large time span, covering 13 years of follow-up of integrated cART and TB care in a cohort of HIV-1/TB co-infected patients from a middle-income country that provides universal access to HIV-1 treatment. We were able to assess mortality outcomes with detailed socio-demographic, clinical, laboratory and treatment data, including the cause of death ascertainment. The CoDe method, used in our analysis is a reliable tool to assess death causality [10]. Existing medical record data was checked for accuracy, active contact with individuals or family members was established and Rio de Janeiro mortality database was consulted in order to ascertain each patient’s vital status. Since this is a cohort study of patients in Rio de Janeiro region, we are limited in our knowledge of how representative this study population is in comparison to HIV-1/AIDS patients cared for at other centers across Brazil. Similarly, to other university hospitals and/or research-based HIV-1 centers in Brazil, INI-Fiocruz provides high standard quality of care. The Southeast part of Brazil, where INI-Fiocruz is located, is the wealthiest part of the country and where there is the largest population of PLHIV. HIV-1 patients cared for at INI-Fiocruz can probably be considered as representative of the HIV-1/AIDS population cared for at Brazilian reference centers in spite of the above-mentioned peculiarities.

## Conclusion

Our results evidence the high morbidity and mortality among patients co-infected with HIV-1 and TB in Rio de Janeiro, Brazil. During the first year following TB diagnosis, mortality was the highest within the initial 3 months, being invasive bacterial infection the major cause of death. CD4 T lymphocyte count ≤50 cells/mm^3^, requirement of mechanical ventilation and presence of disseminated clinical presentation of TB were independently associated with higher mortality risk. In order to successfully intervene in morbidity and mortality among patients co-infected with HIV-1 and TB, it is utterly necessary to address the social determinants of health contributing to the inequitable health care access faced by this population.

## Abbreviations

ART: Antiretroviral Therapy
ARV: Antiretroviral
cART: Combination antiretroviral therapy
CoDe: Coding of Death in HIV
CT: Computed tomography
EFV: Efavirenz
E-MTD®: Amplified *Mycobacterium Tuberculosis* Direct Test
HR: Hazard Ratio
INH: Isoniazid
INI-Fiocruz: Evandro Chagas National Institute of Infectious Diseases – Oswaldo Cruz Foundation
INSTIs: Integrase strand transfer inhibitors
IPT: Isoniazid preventive therapy
LMIC: Low and middle-income countries
MDR-TB: Multi-drug resistant-Tuberculosis
MTB: *Mycobacterium tuberculosis*
MTB-IRIS: *Mycobacterium tuberculosis* immune reconstitution inflammatory syndrome
NNRTI: Non-analogue nucleoside reverse transcriptase inhibitor
NRTI: Nucleoside/nucleotide analogue reverse transcriptase inhibitors
OI: Opportunistic infection
PI: Protease inhibitor
PLHIV: Persons living with HIV
RAL: Raltegravir
RTV: Ritonavir
SQV: Saquinavir
TB: Tuberculosis
